# Potential complications in patients undergoing an ethanol injection into the vein of Marshall

**DOI:** 10.1111/jce.14221

**Published:** 2019-10-15

**Authors:** Kazuo Kato, Akimitsu Tanaka, Shin‐Ichiro Morimoto, Shin Hasegawa, Nobuo Ishiguro, Ryosuke Kametani, Hideo Hattori, Norihisa Shibata

**Affiliations:** ^1^ Department of Cardiology Nagoya Tokushukai General Hospital Kasugai Aichi Japan

**Keywords:** atrial fibrillation, catheter ablation, chemical ablation, complication, ethanol, Marshall vein

## Abstract

**Background:**

Ethanol injections into the vein of Marshall (VOM) (EIM) are considered to be a good therapeutic option for atrial tachyarrhythmias, however, the safety remains to be determined. To elucidate what would affect the safety and potential complications of an EIM, we investigated the anatomical features of the VOM and patient background.

**Methods:**

We performed the EIM before the conventional pulmonary vein isolation for drug‐resistant atrial fibrillation in 88 patients and evaluated the anatomical features of the VOM and their background.

**Results:**

All procedures were completed, however, other than myocardial staining, trivial contrast medium leaked out of the VOM into the pericardial space, that is, extravasation of contrast medium with capillary rupture, during the EIM in 20 patients (22.7%) regardless of the features of the VOM. No pericardial effusions requiring further intervention developed after the extravasation, which resolved by the next day on echocardiography in 18 of those patients. However, two patients who had extravasation other than during the initial contrast injection required additional therapeutic intervention for nonnegligible pericardial effusions. Their body weights were significantly lower and the latter two patients were also small lean women with heart failure and a preserved ejection fraction.

**Conclusions:**

The physical constitution, regardless of the characteristics of the VOM, could be strongly associated with adverse events during the EIM. We must take extreme care in smaller patients with poor compliant hearts during the EIM.

## 1 INTRODUCTION

Ethanol injections into the vein of Marshall (VOM) (EIM) have been recently thought to be one of the therapeutic options for the management of atrial tachyarrhythmias, especially atrial fibrillation (AF) refractory to radiofrequency (RF) ablation.^1^, ^2^, ^3^ This new method has been described as being safe and effective for all patients during the acute phase,^1^, ^2^, ^3^ however, a lethal complication was reported in a fragile patient.^4^

The clinical efficacy of the EIM depends upon the morphology and distribution of the VOM because the EIM pharmacologically may create lesions by injecting ethanol directly into the vein.^1^, ^2^, ^3^ However, what affects the safety and potential complications of the EIM remains to be determined. In this study, we investigated the anatomical features of the VOM and patient background to elucidate the safety of the EIM in relation to those parameters.

## 2 METHODS

### 2.1 Study population

This study included 88 consecutive patients who underwent an EIM and following RF ablation for drug‐resistant AF (paroxysmal: 36, persistent: 23, and longstanding persistent: 29 patients) and were followed for at least 6 months. We defined paroxysmal AF as that terminating spontaneously and lasting for less than 1 week, persistent AF as that lasting for more than 1 week but less than 1 year, and long‐standing persistent AF as that lasting for longer than 1 year. The subjects included 62 men and 26 women with a mean age of 65.4 ± 9.7 years. Written informed consent, including for the EIM, was obtained from each patient before the procedure according to the protocol approved by the Institutional Research Committee.

### 2.2 Ablation procedure

All the procedures were performed under deep sedation with dexmedetomidine, propofol, and buprenorphine, and with esophageal temperature and direct blood pressure monitoring. The activated clotting time was kept at approximately 300 seconds throughout the procedure. All the patients underwent an EIM before the conventional pulmonary vein (PV) isolation (PVI).

The EIM procedures were performed similar to that previously described.^1^, ^2^, ^3^, ^4^ At first, we obtained a coronary sinus (CS) venography by injecting contrast medium via the infusion port of a duodecapolar CS electrode catheter (Abbott, St Paul, MN) without an occlusion balloon to confirm the VOM. Then, we performed a standard transseptal puncture, inserted three sheaths into the left atrium and drew the geometry of the left atrium with a voltage map using a circular catheter and ablation catheter with a 3D mapping system (CARTO; Biosense‐Webster, Diamond Bar, CA, or Ensite NavX; Abbott, St Paul, MN) and placed the circular mapping catheter into the left superior and inferior PVs to record their potentials. After that, we inserted an angiographic catheter for the left internal mammary artery (6 Fr IM; Asahi Intec, Tokyo, Japan) into the CS close to the ostium of the VOM. Through that catheter, a peripheral angioplasty guidewire (0.014″ Cruise; Asahi Intec, Tokyo, Japan) was advanced into the VOM, and an angioplasty balloon catheter (8‐mm length Apex OTW with a 2‐mm nominal diameter and two radiopaque markers on both the distal and proximal ends of the balloon; Boston Scientific, Boston, MA) was then advanced gently over the wire as far as possible into the VOM. Then, following an injection of 0.2‐0.3 mL of radiographic contrast medium for selective VOM venography through the wire lumen of the angioplasty balloon, 2.0 mL of dehydrated ethanol was injected after inflating the balloon with the minimal atmosphere pressure to occlude the VOM, which was commonly less than 4 atms. Then, we pulled the balloon back 8 to 10 mm and repeated the ethanol injection while the distal tip remained inside the VOM. We used a 2‐mL syringe to inject the contrast medium and 1 mL of the ethanol very slowly and gently over more than 1 minute. Throughout the EIM procedure, we carefully checked to confirm whether any contrast medium had leaked out of the VOM or its capillaries into the pericardial space. After the EIM procedure described above, we proceeded to the conventional RF ablation of both ipsilateral PV antra to complete bidirectional block of the PV electrograms.

### 2.3 VOM evaluation

**Figure 1:**
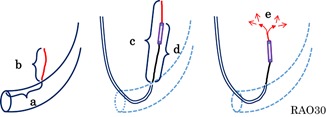
Parameters associated with the VOM features. To evaluate the VOM features, we measured the following parameters: (a) the distance from the CS ostium to the bifurcation of the VOM; (b) the VOM length on the CS venography without an occlusion balloon; (c) the VOM length on the selective VOM‐graphy via the balloon lumen; (d) the length of the balloon advanced from the VOM ostium; and (e) whether or not the contrast medium leaked out into the pericardial space other than any myocardial staining. CS, coronary sinus; RAO, right anterior oblique; VOM, vein of Marshall

To evaluate the anatomical features of the VOM, we measured the distance from the CS ostium to the VOM ostium (a), length of the VOM measured on the CS imaging (b) and that of the direct image taken by injecting contrast medium from the balloon lumen (c), and the depth of the distal tip of the balloon penetrating into the VOM (d) using G‐NAVI version 6.1.0 software (Goodman, Nagoya, Japan). We also checked how many injections of ethanol were administered, and whether or not the contrast medium had leaked out into the pericardial space other than that from myocardial staining, that is, extravasation of the contrast medium with capillary rupture (e) (Figure 1).

### 2.4 Patient follow‐up

We checked if any complications including a trivial pericardial effusion might have occurred using echocardiography soon after all the procedures including after the EIM and PVI had been completed. All patients were hospitalized for at least 3 days after the procedure with continuous monitoring of the ECG and underwent echocardiography at least once on the day after the procedure. We also performed a clinical follow‐up at the cardiology clinic including echocardiography in all patients within 1 month after discharge.

### 2.5 Statistical analysis

Continuous variables are expressed as the mean ± SD. Univariate and multivariate logistic regression analyses were performed to identify the variables associated with the complications. Data analyses were performed using JMP, version 13.0.0 software (SAS Institute, Cary, NC). A *P* < .05 was considered statistically significant.

## 3 RESULTS

### 3.1 Clinical outcome

**Figure 2:**
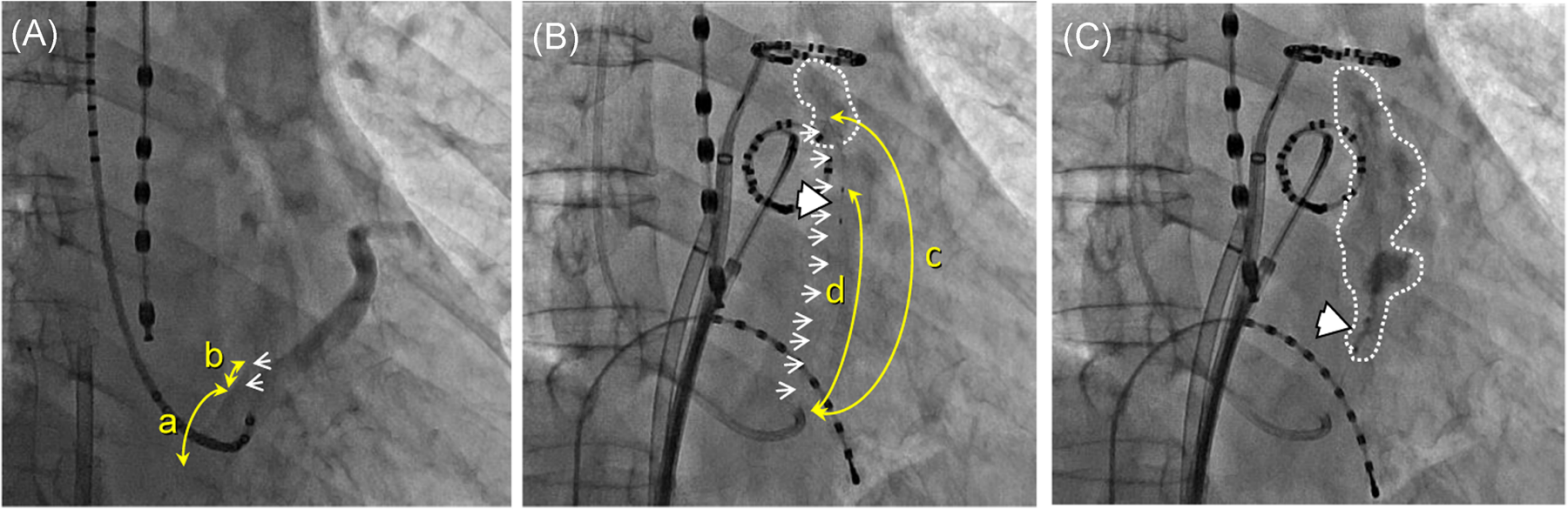
Right anterior oblique (RAO) views of the procedural series of a representative case without any apparent leakage of the contrast medium during the ethanol injection into the vein of Marshall (VOM) (74‐year‐old male, 173.0 cm, 65.6 kg, with no structural heart disease). A, Coronary sinus (CS) venography without an occlusion balloon. B, The initial selective VOM‐graphy via the balloon lumen. C, The image after the third (final) ethanol injection into the VOM. Note that an obscure demarcated myocardial stain is observed around the VOM. The values of the parameters measured regarding the VOM features were as follows: a = 28.01 mm, b = 6.21 mm, c = 70.56 mm, and d = 50.93 mm. (a) through (d) indicate each distance of the actual VOM feature described in the text. The white arrows indicate the image of the VOM by CS venography (A) and the balloon shaft with a selective VOM‐graphy (B). The white arrowheads indicate the position of the balloon. The area surrounded by the white dashed line indicates the area stained by the contrast medium and following ethanol injection

**Figure 3:**
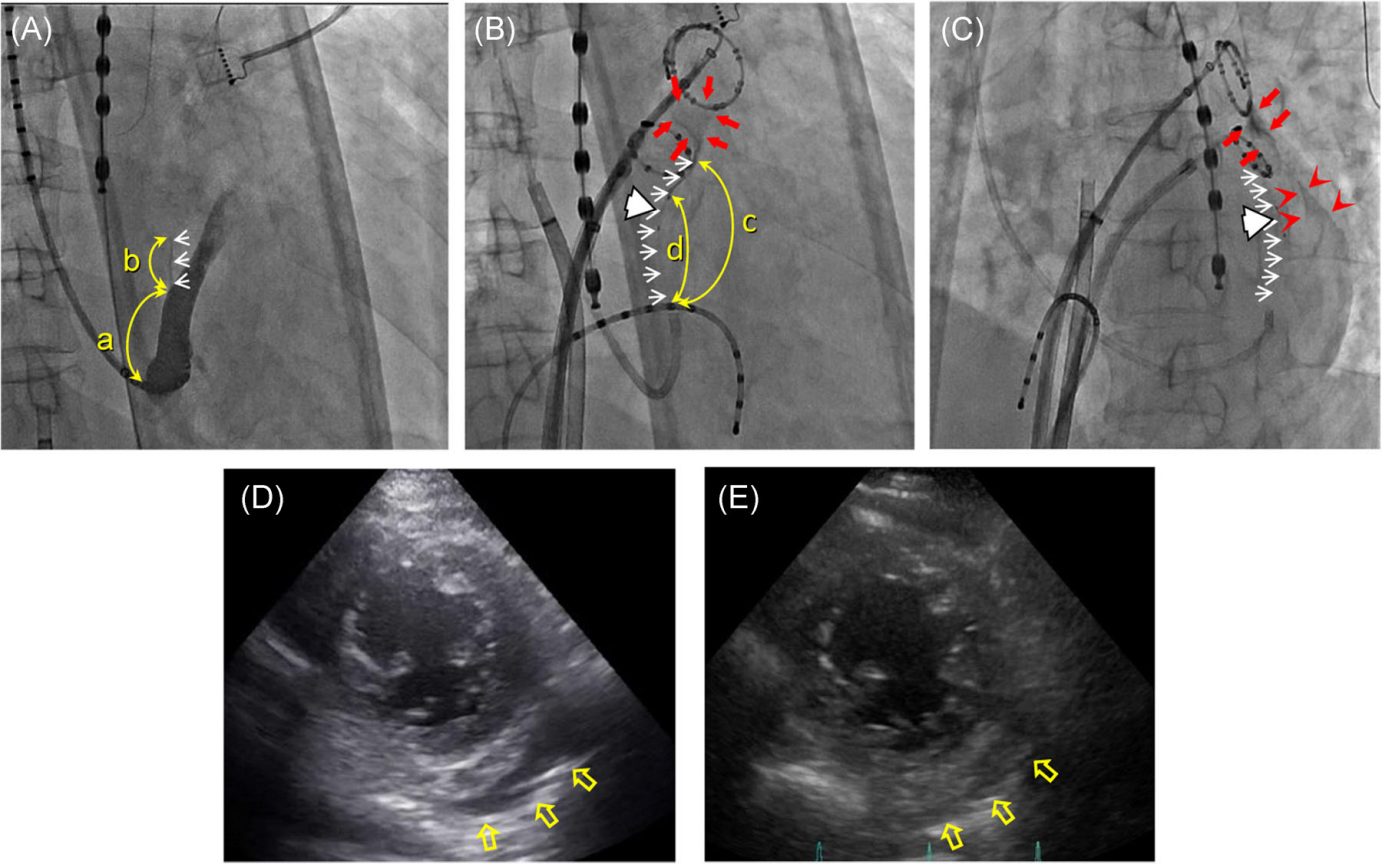
RAO (A,B) and left anterior oblique (LAO) (C) views of the procedural series and echocardiography (D,E) of a representative case with documented apparent leakage of contrast medium into the pericardial space during the initial injection, but the pericardial effusion had almost completely resolved by the next day (63‐year‐old female, 155.0 cm, 52.5 kg, with no structural heart disease). A, CS venography without an occlusion balloon. B,C, The initial selective VOM‐graphy via the balloon lumen in the RAO (B) and LAO (C) views. D,E, Short axis views of the echocardiography examined soon after the session (D) and on the next day (E). Note that fan‐shaped leakage of the contrast medium (red arrows) (B,C) and contrast retention along the cardiac silhouette (red arrowhead) (C) are observed. The pericardial effusion is observed outside of the relatively thick adipose layer (yellow empty arrows) soon after the session despite no deterioration of her hemodynamic condition (D), which becomes mostly resolved by the next day (E). The values of the parameters measured regarding the VOM features were as follows: a = 25.9 mm, b = 14.51 mm, c = 41.74 mm, and d = 30.49 mm. Abbreviations, (a) through (d), the white arrows, and white arrowheads indicate the same as in Figure 2

All the procedures including the EIM and following PVI were completed in all 88 patients. The area around the VOM was stained with the contrast medium and the following ethanol injection in all patients. Most of the stained lesions were obscurely demarcated (Figure 2), however, in addition to staining, trivial contrast leaked out of the VOM capillaries into the pericardial space, that is, extravasation of the contrast medium with capillary rupture during some of the EIMs, in 20 patients. Fifteen out of those 20 patients had extravasation with capillary rupture during the initial injection and the remaining five patients during the following EIM. Very slight pericardial effusions were observed by echocardiography soon after the procedure in some patients, but none developed into any adverse hemodynamic conditions, nor required further intervention other than the two patients stated below. The pericardial effusions were observed to be almost completely resolved by the next day (Figure 3), and they were discharged without any sequela, and there were no adverse events during the follow‐up in 86 patients.

**Figure 4:**
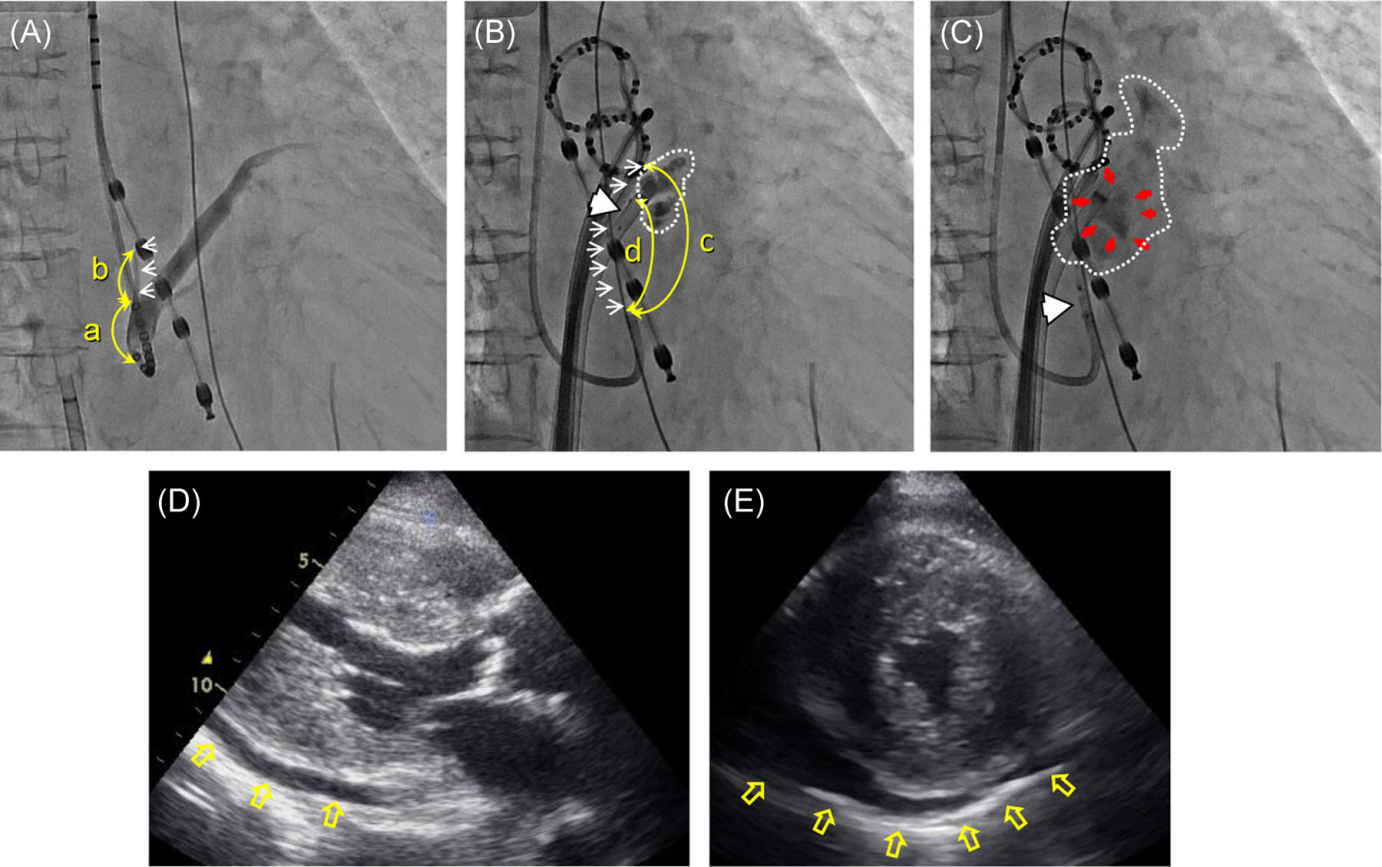
RAO views of the procedural series (A‐C) and the echocardiography (D,E) of the case with documented manifest leakage of the contrast medium into the pericardial space during the third injection that remained for a few days (79‐year‐old female, 147.0 cm, 47.0 kg, with hypertrophic obstructive cardiomyopathy). A, CS venography without an occlusion balloon. B, The initial selective VOM‐graphy via the balloon lumen. C, The third selective VOM‐graphy and the following ethanol injection via the balloon lumen. D,E, Long and short‐axis views of the echocardiography examined soon after the session (D) and on the next day (E). Note that a well‐demarcated contrast retention (red arrows) inside of the obscure demarcated myocardial stain surrounded by the white dashed line during the third injection of contrast medium is observed (C). The pericardial effusion soon after the procedure (D) had increased gradually by the next day (E) (yellow empty arrows), so she underwent a prolonged hospitalization. The values of the parameters measured regarding the VOM features were as follows: a = 17.45 mm, b = 14.42 mm, c = 48.43 mm, and d = 39.58 mm. Abbreviations, (a) through (d), the white arrows, and white arrowheads indicate the same as in Figure2

**Figure 5:**
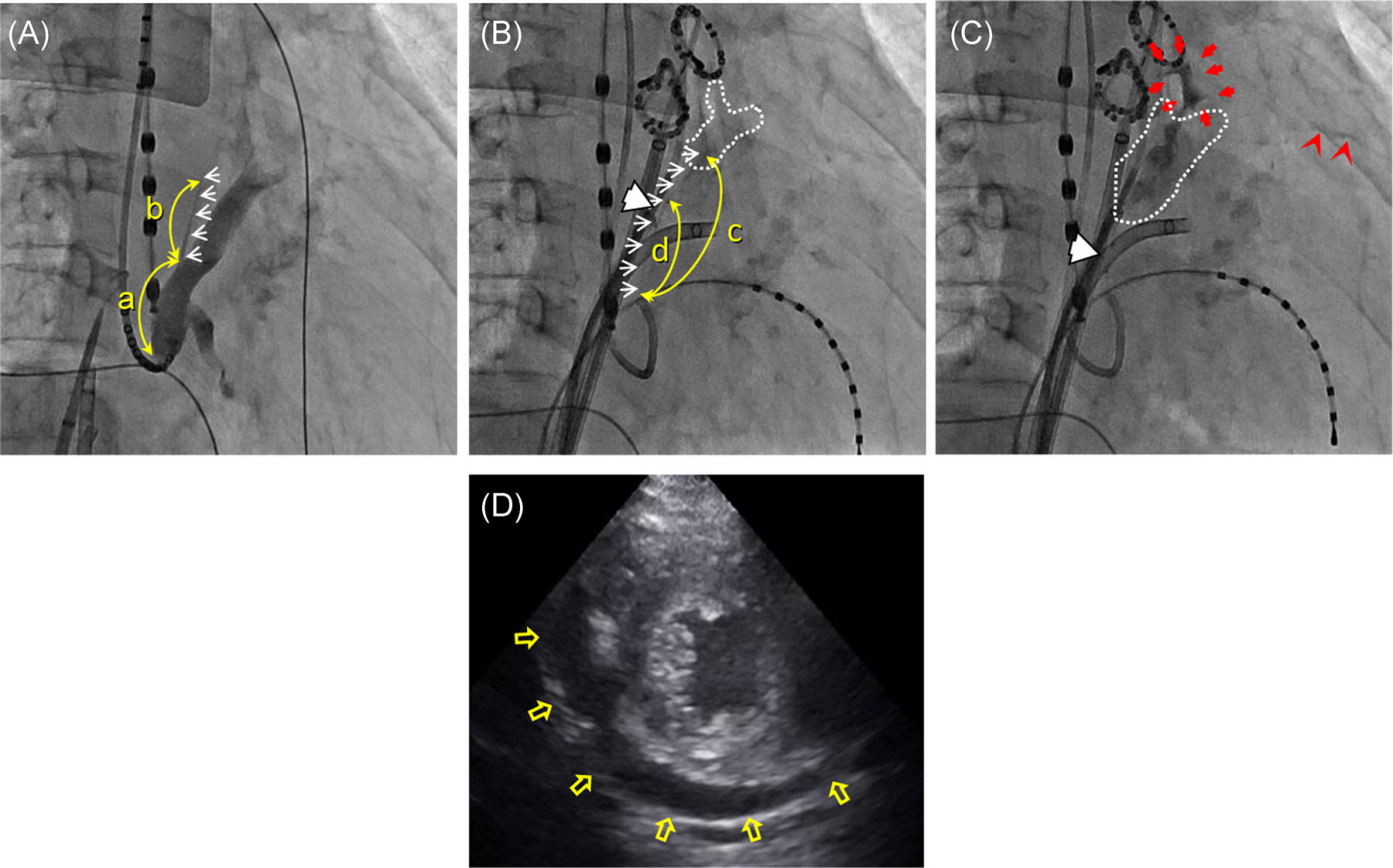
RAO views of the procedural series (A‐C) and echocardiography (D) of the case with documented manifest leakage of the contrast medium into the pericardial space during the third injection, which required pericardiocentesis (57 year‐old female, 151.0 cm, 49.4 kg, with heart failure with a preserved ejection fraction on hemodialysis for 20 years). A, CS venography without an occlusion balloon. B, The initial selective VOM‐graphy via the balloon lumen. C, The third selective VOM‐graphy and following ethanol injection via the balloon lumen. D, Short axis view of the echocardiography examined soon after the session. Note that a well‐demarcated fan‐shaped leakage and contrast retention (red arrows) outside of the obscure demarcated stain surrounded by the white dashed line and contrast retention along the cardiac silhouette (red arrowheads) are observed during the third injection of the contrast medium and following ethanol injection (C). Pericardiocentesis was required to manage the pericardial effusion (yellow empty arrows) (D). The values of the parameters measured regarding the VOM features were as follows: a = 27.25 mm, b = 20.16 mm, c = 42.47 mm, and d = 25.92 mm. Abbreviations, (a) through (d), the white arrows, and white arrowheads indicate the same as in Figure2

As stated above, two out of the five patients who had extravasation of the contrast medium with capillary rupture other than during the initial EIM, required an additional therapeutic intervention. One who underwent an additional procedure for extravasation with capillary rupture was a 79‐year‐old female (height 147 cm, weight 47.0 kg) with hypertrophic obstructive cardiomyopathy (HOCM) that provoked heart failure with a preserved ejection fraction (HFpEF) and paroxysmal AF (Figure 4). The left ventricular ejection fraction and left atrial diameter on echocardiography were 89% and 42 mm, respectively. The extravasation with capillary rupture occurred during the third EIM and the ultrasound monitoring revealed a modest pericardial effusion, however, the blood pressure was maintained without pericardial centesis during the procedure. The pericardial effusion remained for a few days, so she underwent a prolonged hospitalization to receive further observation. Early recurrence of AF occurred during her hospitalization, and she was discharged after pacemaker implantation for atrioventricular synchronous pacing to manage her HOCM. The other patient was a 57‐year‐old female (height 151 cm, weight 49.4 kg) with persistent AF and HFpEF who depended on hemodialysis for 20 years (Figure 5). Her left ventricular ejection fraction and left atrial diameter on echocardiography were 56% and 51 mm, respectively. The extravasation with capillary rupture occurred during the third EIM and required a pericardial centesis to drain a hemopericardium after the completion of the PVI. However, after intensive care, she developed nonocclusive mesenteric ischemia following transient hypotension leading to multiple systemic organ failure and death 2 days after the procedure.^4^

### 3.2 Comparison of the patient background

**Figure 6:**
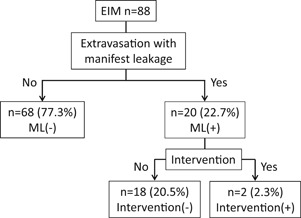
Patient distribution according to the adverse events following the EIM. EIM, ethanol injection into the vein of Marshall; ML, extravasation with manifest leakage

We classified all the cases into two groups according to the presence of manifest extravasation of the contrast medium with capillary rupture to investigate the factors associated with the adverse events during the EIM: ML(−) group, cases without any manifest leakage of the contrast medium into the pericardial space other than myocardial staining, or did not require any procedure‐related intervention during the periprocedural period; and ML(+) group, cases with manifest leakage of the contrast medium from the VOM capillaries into the pericardial space independent of any myocardial staining. Furthermore, we divided the cases in the ML(+) group with a procedure‐related intervention into the following: Intervention(−) group, cases with a trivial pericardial effusion soon after the session that nearly resolved by the next day; and Intervention(+) group, cases with a pericardial effusion that required a prolonged hospitalization or affected the hemodynamic condition (Figure 6).

In the univariate analyses, there were no significant differences in the type of fibrillation, age, echocardiographic parameters, comorbidities, or features of the VOM between the ML(−) and other groups. On the other hand, the parameters regarding the physical constitution were associated with a manifest leakage of the contrast medium in addition to myocardial staining. Manifest leakage of the contrast medium during the initial EIM was frequently observed in the Intervention(−) group, however, none of the cases in the Intervention(+) group had manifest extravasation of the contrast medium with the capillary rupture during the initial EIM. In the multivariate analyses, the body weight was the only significant parameter that differed between the ML(−) and ML(+) groups (Table 1).

**Table 1:** Patient characteristics and variables associated with adverse events following the EIM

	ML(−) (n = 68)	ML(+) (n = 20)		Univariate (*P* value)			Multivariate (*P* value)
		Intervention (−) (n = 18)	Intervention (+) (n = 2)	ML(−) vs ML(+)	ML(−) vs Intervention (−)	ML(−) vs Intervention(+)	ML(−) vs ML(+)
ParAF/PerAF/LSAF	28/16/24	7/6/5	1/1/0	.5324	.6808	.3950	⋯
Male/Female	50/18	12/6	0/2	.2528	.5688	.0232	.6272
Age, y	65.5 ± 9.3	64.8 ± 11.2	68.0 ± 15.6	.8756	.7826	.6944	⋯
Height, cm	166.0 ± 8.3	162.3 ± 8.9	149.0 ± 2.8	.0213	.0941	.0010	⋯
Bodyweight, kg	67.6 ± 12.6	60.4 ± 9.7	48.2 ± 1.7	.0049	.0202	.0111	.0084
BMI, kg/mm^2^	24.4 ± 3.5	22.9 ± 2.9	21.7 ± 0.1	.0483	.0815	.2488	⋯
Echocardiography							
EF, %	64.4 ± 9.7	62.1 ± 9.9	72.5 ± 23.3	.5987	.3606	.2203	⋯
LAD, mm	41.8 ± 5.7	41.1 ± 7.3	46.5 ± 6.4	.9039	.6512	.2155	⋯
Comorbidities							
CHADS2	1.2 ± 0.8	1.4 ± 0.9	2.0 ± 1.4	.3272	.4950	.2381	⋯
Hypertension	37 (54.4)	6 (33.3)	2 (100.0)	.2560	.1090	.1224	⋯
Diabetes	17 (25.0)	5 (27.8)	1 (50.0)	.6579	.8114	.4557	⋯
CAD	11 (16.2)	1 (5.6)	0 (0.0)	.1605	.2069	.4046	⋯
CKD	11 (16.2)	3 (16.7)	1 (50.0)	.6937	.9601	.2782	⋯
Number of EIM	2.7 ± 1.0	3.0 ± 1.0	4.0	.0964	.2213	.0689	.1001
Leakage on initial EIM	0	15 (83.3)	0 (0.0)	<.0001	<.0001	⋯	⋯
VOM characteristics, mm							
a	28.1 ± 10.6	28.1 ± 9.1	22.4 ± 6.9	.8288	.9930	.4167	⋯
b	13.9 ± 9.7	14.8 ± 11.1	17.3 ± 4.1	.6518	.7375	.6495	⋯
c	60.5 ± 34.2	59.8 ± 29.6	65.8 ± 6.7	.9907	.9363	.8301	⋯
d	27.4 ± 18.4	30.8 ± 17.0	46.2 ± 12.6	.2927	.4852	.2002	⋯

*Note:* Data are presented as the n (%) or mean ± SD.

Abbreviations: AF, atrial fibrillation; BMI, body mass index; CAD, coronary artery disease; CKD, chronic kidney disease; EF, ejection fraction; EIM, ethanol injections into the vein of Marshall; LAD, left atrial diameter; LSAF, longstanding persistent AF; ML, extravasation with manifest leakage; ParAF, paroxysmal AF; PerAF, persistent AF; VOM, vein of Marshall.

## 4 DISCUSSION

Chemical ablation using an ethanol injection through a vessel has been widely accepted clinically and also applied to cardiac diseases.^5^ This method was first described for ventricular tachycardia in 1989 by injecting ethanol into the culprit coronary artery, of which the complications were mainly caused by direct myocardial injury from the ethanol provoking atrioventricular block.^6^, ^7^ No significant worsening of the systolic function nor dissections or perforations of the coronary artery related to catheter manipulation were reported, but the authors mentioned that ventricular dysfunction might be potentially aggravated and injuries to the coronary artery could also occur during the procedure.^6^, ^8^

In 2009, Valderrábano et al^1^, ^2^ reported the feasibility of applying an ethanol infusion in the coronary vein especially in the VOM. Since then, many papers have been published regarding this new method and no serious complications including vessel injury have occurred and all the procedures have been performed very safely. However, we experienced 20 out of 88 cases with extravasation associated with capillary rupture regardless of the features of the VOM. No pericardial effusions requiring further intervention developed after the extravasation, which resolved by the next day on echocardiography in 18 of those patients, however, two patients who had extravasation other than during the initial contrast injection, required an additional therapeutic intervention for nonnegligible pericardial effusions. Their body weight was significantly lower, and the latter two patients were also small lean women with HFpEF.

Since we experienced those latter two cases with major complications, observed especially in fragile patients within 1 year from when we first performed an EIM procedure, we have been injecting the ethanol more slowly and gently ever since. However, fluoroscopically we experienced contrast medium leaking out into the pericardial space in addition to myocardial staining in 17.0% of cases (15 of 88) during the first ethanol injection regardless of the characteristics of the VOM, but none required any pericardial centesis and the pericardial effusions had almost completely resolved after the procedure on ultrasound monitoring. This phenomenon has occurred with a similar frequency even in the latest phase.

We previously reported the histopathology after an EIM in which the intima of the venule was compressed and injured adjacent to the lacerated vein.^4^ The venule wall is composed of three layers like the arteriole wall, but the middle layer of the venule is thinner because of fewer elastic fibers, unlike the arterioles, while the coronary veins on the surface of the epicardium are surrounded by rich fat. Namely, the venule might be more porous and easily lacerated but could be protected against continuous bleeding by the fat around it. Several patients experienced manifest leakage of the contrast medium into the pericardial space with capillary rupture but the majority of the pericardial effusions resolved by the next day, suggesting that the epicardial fat around the coronary vein could have provided hemostasis and might have absorbed the leaked contrast medium and bleeding if the damage to the venule leading to the leakage during the initial injection was trivial. On the contrary, in physically small lean women with a poor compliant heart as we experienced, their venules and the epicardial fat around them might be too fragile to restore their serious conditions such as requiring pericardial centesis regardless of the features of the VOM.

## 5 STUDY LIMITATIONS

First, this was a retrospective cohort study that included very few complications. Therefore, we could not perform multivariate analyses to determine the predictors of the Intervention(+) group, which required additional intervention, and there might not have been an adequate statistical power even in the univariate analyses. However, all the cases in the Intervention(+) group were physically small women and the multivariate analyses showed that the body weight was the only significant predictor of the ML(+) group, which included the Intervention(+) group, suggesting that leanness might be one of the important risk factors of the extravasation of the contrast medium with capillary rupture leading to adverse events. Furthermore, out of all the patients, only two cases suffering from HFpEF required intervention. They might have had a decreased heart compliance with an increased central venous pressure, which could have been responsible for the serious complications. It was not possible to statistically analyze the correlation between the existence of HFpEF and the complications, however, it might still remain necessary to clarify whether the heart compliance could affect the results.

Second, we experienced two cases with complications during the early phase even when injecting the ethanol very slowly (1 mL over more than 1 minute). After that, we fortunately did not experience any further serious complications since we began performing the ethanol injection using the same size syringe but by delivering the ethanol more slowly and gently than before. However, we encountered several patients with trivial extravasation associated with a capillary rupture that resolved without any further intervention. We did not measure the accurate pressure using a manometer during the injections, and therefore, we could not objectively determine the threshold related to the serious complications.

Finally, the extravasation of the contrast medium with capillary rupture was determined visually with fluoroscopy, so we might have missed an imperceptible extravasation. Further, the above hypotheses were based upon the pathophysiological findings observed in only one case.^4^ Further experience is required to clarify the mechanisms of the complications of the EIM to perform a safer EIM.

Considering all the above findings, we think the physical constitution and underlying heart disease regardless of the characteristics of the VOM would be the most important factors for complications related to the EIM in addition to how gently the EIM is performed. We have to keep in mind that the EIM could compress the vessel intima leading to injury to its wall, and we should refrain from any hasty injection into the vein especially in fragile and small lean patients with underlying heart disease and a poor compliant heart.

## CONFLICT OF INTERESTS

The authors declare that there are no conflict of interests.

## AUTHOR CONTRIBUTION

KK performed the conception, design, and data collection. S‐IM and HH analyzed the histopathological findings. AT, NS, SH, NI, and RK performed the data collection.
